# Book Notices

**Published:** 1889-11

**Authors:** 


### Book Notices.

SWEEDISH MOVEMENT AND MASSAGE TREATMENT, A MANUAL of Instruction by Prof. H. Nisseu, Director of the Swedish Heath Institute, Washington, D. C. F. A. Davis, Publisher, 1889., pp 128. Price $1.

This little book explains the Swedish movement and treatment. It gives the indications for treatment and how to apply it. The book shows how operators are to be educated and trained and prescribed by physicans. It is a handy little book for any one who wishes to post himself scientifically on the subject of Swedish movement and massage.

---

LECTURES ON BRIGHT'S DISEASE, By Robt. Saundly, M. D., Edin. Fellow of the Royal Physicians, London. E. B. Treat, Publisher, N. Y., 1889.

This book has fifty illustrations and 290 pages. The author is well known and is good authority on the subject treated of in the book. While there is not a great deal that is specially new in the work it is well up to date and thorough in all the details of the subject.

---

THE FUNCTIONS AND DISORDERS OF THE REPRODUCTIVE ORgans in Childhood, Youth, Adult Age, and Advanced Life, By William Acton, M. R. C. S., of Paris, France, P. Blakiston, Son & Co., Philadelphia, 1888.

This is certainly a popular book. It has already run through six large editions, this being the seventh. It contains 260 pages well printed.

The subject of this book has been left too much to charlatans. The "errors and indecretions of youth" is a familiar advertisement that has caught many a young man. Such a work as the one under consideration does credit to the head and heart of the distinguished author, and its popularity among the profession may be looked upon as an indication that the snbject is being reclaimed from the hands of quackery and given its true place in legitimate medicine.

ELECTRICITY AND THE METHODS OF ITS EMPLOYMENT IN REMOVING Superfluous Hairs and Other Facial Blemishes. By Plym S. Hayes, A. M. M. D., Chicago, 1889. W. T. Keener, Publisher, Chicago.

This is a small book of only 123 pages, but it contains all the information needed by an intelligent practitioner to enable him to successfully employ electrolysis in the removal of superfluous hairs, moles, nævi, etc.

This process has been known for thirteen years. It is now perfected and established.

---

TRANSACTIONS of the Texas State Medical Association. Twenty-first annual session, held at San Antonio, Texas, April, 1889. Eugene von Boeckmann, Austin, 1889.

"Many interesting reports are made and well put together, comprising a neat volume of over three hundred pages, among which, in the section on pathology, is an account of two cases of leprosy, in which the non-contagious nature of the disease is advocated. But one important fact is overlooked—that leprosy sometimes appears after an interval of from five to twenty years, and that those with whom the lepers may come in contact may develop the disease in future years. The best authority upon the subject holds that leprosy is contagious.—Medical Bulletin."

Dr. Dock thinks differently.

